# Algorithm of the choice of nutritional therapy in infants with food allergy

**DOI:** 10.1186/2045-7022-3-S3-P111

**Published:** 2013-07-25

**Authors:** MY Belitskaya, SN Denisova, TB Sentsova, VA Revyakina, AA Trofimova, EV Pavlovskaya

**Affiliations:** 1 Russian National Research Medical University, Moscow, Russian Federation; 2 Research Institute of Nutrition, Russian Academy of Medical Sciences, Moscow, Russian Federation

## Background

Cow milk protein sensitization is common in bottle-fed infants. It is known about immunological cross-reaction for cow and goat milk protein. The problem of nutritional correction in infants with cow milk protein allergy in lack of breastfeeding has a high clinical importance.

## Methods

We examined 50 formulae fed infants 1-12 months old with food allergy. The examination included a collection of allergy history, clinical evaluation and specific methods – the measurement of the coprofiltrate levels of total IgE and allergen-specific IgE to the cow milk protein and casein. The level of total IgE, cow and goat milk proteins IgE allergen-specific antibodies in coprofiltrates were measured by immunoenzyme method using the spectrophotometer “Sunrise” (Belgium), with test-systems “Allergopharma” and “Dr. Fooke” (Germany).

The nutritional therapy of the food allergy in infants consists in replacement of cow milk protein formula on the formula based on the goat milk. The estimation of the efficiency of therapy based on dynamics of the clinical symptoms of the disease and decrease of the serum levels of total IgE and allergen-specific IgE to the cow milk proteins.

## Results

Use of adapted formula based on the goat milkin the complex anti-allergic therapy resulted in positive clinical effect in 37/50 (74%) infants. The remission of atopic dermatitis and gastrointestinal allergy was observed on 10^th^ -20^th^ day of the treatment and was accompanied by decrease of the serum levels of total IgE and allergen-specific IgE to the cow milk proteins.

## Conclusion

The beneficial clinical effect was observed in 74% infants with skin and gastrointestinal forms of the food allergy during the use of adapted formula based on the goat milk. The detection of general IgE and allergen-specific IgE antibodies to cow’s milk protein and goat’s milk protein in coprofiltrates makes it possible to optimise the diet therapy for infants with cow milk protein allergy.

## Disclosure of interest

None declared.

